# Inflammatory bowel disease and prostate cancer risk: A systematic review

**DOI:** 10.1080/2090598X.2020.1761674

**Published:** 2020-05-19

**Authors:** Anoud Haddad, Mohammed Qussay Al-Sabbagh, Hashim Al-Ani, Abdel Muez Siyam, Emad Aborajooh, Takehiro Iwata, Shoji Kimura, Shahrokh F. Shariat, Mohammad Abufaraj

**Affiliations:** a Division of Urology, Department of Special Surgery, Jordan University Hospital, the University of Jordan, Amman, Jordan; b Department of Surgery, Faculty of Medicine, Mutah University, Kerak, Jordan; c Department of Urology, Medical University of Vienna, Vienna, Austria; d Department of Urology, Okayama University Graduate School of Medicine, Dentistry and Pharmaceutical Sciences, Okayama, Japan; e Department of Urology, Jikei University School of Medicine, Tokyo, Japan; f Department of Urology, Weill Cornell Medical College, New York, NY, USA; g Department of Urology, University of Texas Southwestern Medical Center, Dallas, TX, USA; h Karl Landsteiner Institute of Urology and Andrology, Vienna, Austria; i Institute for Urology and Reproductive Health, Sechenov University, Moscow, Russia

**Keywords:** Prostate cancer, inflammatory bowel disease, Crohn’s disease, ulcerative colitis, cancer risk

## Abstract

**Objective**: To evaluate the risk of prostate cancer (PCa) in patients with inflammatory bowel disease (IBD), focussing on ulcerative colitis (UC) and Crohn’s disease (CD) separately.

**Methods**: A systemic search was carried out using PubMed and Web of Science databases following the Preferred Reporting Items for Systematic Reviews and Meta-Analyses (PRISMA) guidelines. We retrieved a total of 349 articles. All the articles were in the English language and investigated the incidence of PCa in patients with IBD.

**Results**: Nine studies met our inclusion criteria, with a total of 205 037 men. Two studies reported an increase in the risk of PCa in men with IBD in general. Five other studies reported an increased risk of PCa in men with UC or with CD specifically. On the other hand, two studies reported a decreased risk of PCa in patients with UC and patients with IBD treated with aminosalicylates.

**Conclusions**: While men with UC appear to have higher risk of developing PCa, data on patients with CD are inconclusive. Therefore, patients with UC may benefit from earlier PCa screening. Our findings confirm a complex interplay between IBD and PCa, including factors such as genetic predisposition, systemic inflammation and treatment effects. The modulatory effect of treatment strategies for IBD on the development and progression of PCa might be of clinical significance.

**Abbreviations:** CD: Crohn’s disease; CRP: C- reactive protein; FOLH1: folate hydrolase 1; GIT: gastrointestinal tract; IBD: inflammatory bowel disease; IL-6: interleukin 6; NOS: Newcastle–Ottawa Scale; PCa: prostate cancer; PRISMA: Preferred Reporting Items for Systematic Reviews and Meta-Analyses; PSMA: prostate-specific membrane antigen; UC: ulcerative colitis.

## Introduction

Inflammatory bowel disease (IBD) is a chronic, idiopathic, inflammatory status of the bowel comprising two major entities; ulcerative colitis (UC) and Crohn’s Disease (CD) [1]. The highest reported prevalence of IBD is observed in Europe and North America, with an estimated incidence of 505 per 100 000 in Norway and 322 per 100 000 in Germany for UC and CD, respectively. Although the incidence of IBD is stable in highly prevalent regions, the trends have been increasing since 1990 in some newly industrialised countries in Africa and southern America, resulting in increased overall global incidence [2].

Patients with IBD are at increased risk of developing various types of cancers [3–5]. The chronic inflammatory status of the gastrointestinal tract (GIT) predisposes these patients to a higher risk of developing various GIT malignancies [3,5]. There is, additionally, increasing evidence that the body’s chronic inflammatory response and the systemic treatment of IBD increases the risk of other extra-intestinal tumours, such as skin and haematopoietic malignancies [3,4].

Several investigators have evaluated the association between IBD and urological malignancies, such as bladder cancer [3,4]. The association between IBD and the risk of developing prostate cancer (PCa) remains unclear. We hypothesised that IBD would be associated with an increased risk of developing PCa. Therefore, we evaluated the characteristics and risks of PCa in patients with IBD focussing on UC and CD separately.

## Materials and methods

### Search strategy

A systematic literature search was conducted using PubMed and Web of Science databases, on 1 October 2019. We retrieved all published articles written in the English language that investigated the association between IBD and PCa risk. The following string terms were used: prostate cancer and (‘Inflammatory bowel disease’ or ‘ulcerative colitis’ or ‘Crohn’s disease’) with an overall result of 349 articles. This process was carried out by two independent reviewers who screened study titles and abstracts in order to assess and exclude irrelevant publications.

### Inclusion criteria

Studies were only included if they reported on the prevalence/incidence of PCa in patients with IBD, or an association between IBD and PCa, only if including more than five cases of PCa.

### Quality assessment

The quality of each individual study was assessed by two reviewers independently according to the Cochrane handbook [6]. The Newcastle–Ottawa Scale (NOS) was used to assess the quality of the included studies [7]. The scale focusses on three factors: Selection (1–4), Comparability (1–2) and Exposure (1–3). The total score ranges from 0 (lowest) to 9 (highest). The presence of confounders was determined by consensus and review of the literature.

### Data extraction

Full-text articles were reviewed by two other reviewers independently to extract the required data based on pre-specified Excel sheets. The first author, year of publication, sample size, and the incidence of PCa in patients with IBD amongst other demographic data in all eligible studies were collected. All discrepancies regarding data extraction were resolved by consensus.

## Results

**Figure 1.**
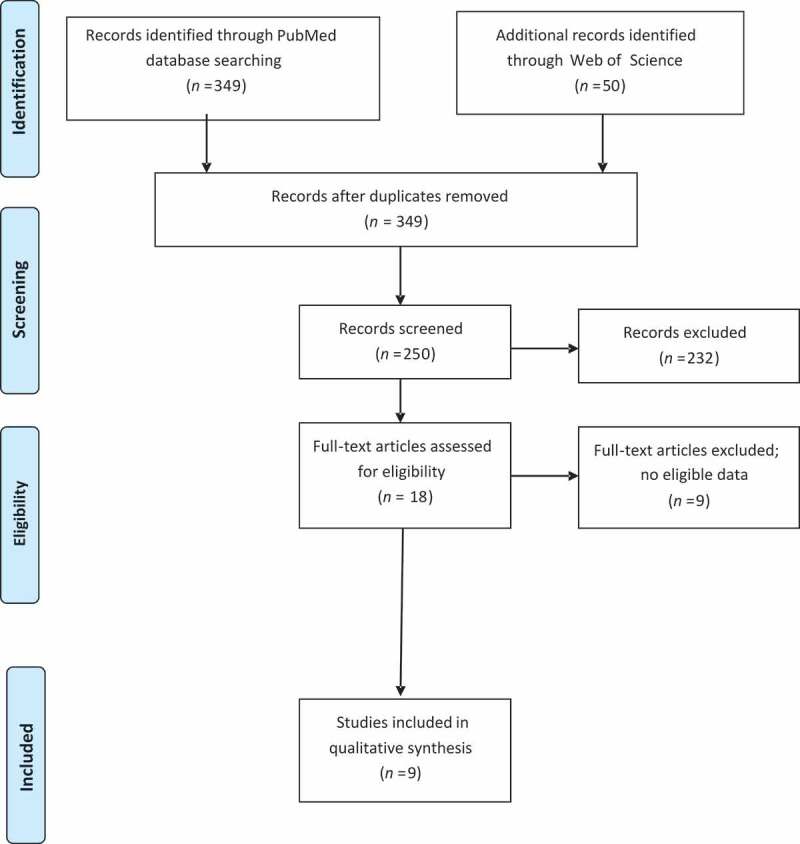
PRISMA flowchart

The Preferred Reporting Items for Systematic Reviews and Meta-Analyses (PRISMA) checklist was adopted in conducting this systematic review (Figure 1). Our protocol was registered in the international prospective register of systematic reviews database (International Prospective Register of Systematic Reviews [PROSPERO]: CRD 42,019,146,910).

After the initial search, a total of 349 studies were identified; out of which 331 studies were excluded after title and abstract assessment. An additional nine studies were excluded based on a full-text review. Finally, nine studies were included for qualitative analysis.

### Characteristics of the included studies

Seven reports evaluated the incidence of various malignancies, including PCa, in patients with IBD. One study reported on the risk of developing PCa in patients with IBD and two other studies evaluated cancer risks in patients with CD and UC, respectively. Table 1 [8–16] summaries the characteristics of the included studies.

**Table 1.** Characteristics of the included studies

Reference	Country	Patients, *n*	IBD patients, *n*	PCa patients, *n*	Mean/median follow-up, years	Diagnosis	Comments
Burns et al., 2019 [8]	USA	10,339	1033	715	5.6	IBD	Increased risk of clinically significant PCa for men with IBD
So et al., 2017 [13]	China	2621	893^a^	8	10^b^	UC	There was an increased risk of PCa in UC patients
Khan et al., 2017 [9]	USA	63,759	59,916	204	22 or 24^c^	IBD	PCa had higher IRs in the elderly IBD subgroup compared with the age-matched SEER database.
Jung et al., 2017 [14]	Korea	15,291	15,291	19	2.14	IBD	A significant increased risk of PCa was found in UC patients but not in CD patients.
Wilson et al., 2016 [15]	UK	39,294	19,647	79	6.4	IBD	A significant reduction in PCa risk was observed in IBD patients prescribed aminosalicylates
Jussila et al., 2013 [10]	Finland	21,964	2634^d^	150	10.8	UC	A reduced frequency of PCa was observed among male UC patients
Jess et al., 2013 [16]	Denmark	2325	1083^d^	29^e^	15	UC	An excess risk of PCa in patients with UC
Hemminki et al., 2009 [11]	Sweden	21,788	21,788	152	__	CD	Increased risks of cancer were observed for common sites, such as the prostate (SIR 1.19)
Hemminki et al., 2008 [12]	Sweden	27,656	27,656	277	__	UC	Common sites for malignancy, such as the prostate (SIR 1.14) were in excess

CD: Crohn’s disease; IBD: inflammatory bowel disease; SIR: standardised incidence ratio; UC: ulcerative colitis.

^a^UC patients only.

^b^10 years for UC and 8 years for CD.

^c^24 months for elderly and 22 months for the younger population.

^d^Male patients only.

^e^All male organs’ malignancies.

There were five retrospective studies [8–12] comprising 145 506 patients and four prospective studies [13–16] comprising 59 531 patients. Of these, two studies enrolled patients from the USA, five from Europe, and two from Asia.

### Outcomes

#### IBD

This systematic review found that men with IBD, in general, seem to have a higher risk of developing PCa than the general population. An increased risk of PCa in men with IBD was reported in two studies [8,9]. It is worth mentioning that, Wilson et al. [15] reported a lower risk of PCa in patients with IBD treated with aminosalicylates.

#### UC

An increased risk of PCa in men with UC was reported in four studies [12–14]. One study, on the other hand, reported a lower risk of PCa in men with UC [10].

#### CD

Only one study reported an association between PCa and CD, where an increased risk of PCa was noted [11]. A formal meta-analysis on the risk of PCa in patients with IBD was not performed owing to the limited number of publications and the heterogeneity of the data.

Table 2 [8–16] summarises the quality assessment, selection, comparability, and outcome in each of the included studies.

**Table 2.** NOS of the eligible studies that analyses the association between IBD and prostate cancer

Reference	Selection	Comparability	Outcome	Total
Burns et al., 2019 [8]	★★★	★★	★★★	8
So et al., 2017 [13]	★★★★	★★	★★	8
Khan et al., 2017 [9]	★★★★	★★	★★	8
Jung et al., 2017 [14]	★★★★	★★	★	7
Wilson et al., 2016 [15]	★★★★	★★	★★	8
Jussila et al., 2013 [10]	★★★★	★	★★	7
Jess et al., 2013 [16]	★★★★	★★	★★	8
Hemminki et al., 2009 [11]	★★★	★★	★★	7
Hemminki et al., 2008 [12]	★★★	★★	★★	7

## Discussion

### Principle findings

In the present systematic review, we aimed to investigate whether patients with IBD are at a higher risk of developing PCa. Overall, patients with IBD do appear to have a higher risk of developing PCa. This is especially evident in patients with UC rather than patients with CD. This association was established in six studies. One report showed an increased risk of PCa in patients with CD [11]. Two other reports appeared to have contradictory findings; Jussila et al. [10] reported that UC male patients’ risk of PCa was actually slightly less than the general population, while Wilson et al. [15] did not identify a significant association between IBD treated with aminosalicylates and PCa risk.

### Mechanisms of the association between IBD and PCa risk

While several investigators investigated the potential association between IBD and PCa risk, the impact of IBD on disease risk remains unclear [9,11,15,16]. The postulated association can be explained by various mechanisms; however, it is complex and multifactorial.

### Genetic background

Some authors postulated that there are mutual genetic susceptibility genes between IBD and PCa [8,17]. This finding is consistent with the fact that many autoimmune and neoplastic diseases have a common genetic background and certain genetic aberrations may confer increased risk of developing malignancies. A noteworthy example is the folate hydrolase 1 (FOLH1)/prostate-specific membrane antigen (PSMA), which appears to be upregulated in both IBD and PCa [18–21]. In fact, FOLH1/PSMA overexpression was found to be correlated with biochemical recurrence and metastasis in patients with PCa [22]. Additionally, FOLH1/PSMA inhibition resulted in tumour growth regression and size decrease in animal models [22]. Inhibiting FOLH1/PSMA also resulted in mitigation of colonic inflammation in IBD mice models [18], indicating a significant pathophysiological role for FOLH1/PSMA in both diseases.

### Inflammatory state

In addition, the risk of PCa may be associated with IBD due to the inherent inflammatory process [23–25]; with a regional and/or systematic effect. For example, the rectum is frequently involved in IBD (common in CD, always in UC), and rectal inflammation may directly or indirectly be related to prostate pathologies, such as prostatitis. Chronic prostatitis has been suggested to eventually induce tumorigenesis within the prostate via DNA damage and oxidative stress [23–26]. Prostatitis may also be related to microbiome translocation via the inflamed bowel to the circulation, where they may ‘home’ to the prostate, among other tissues, and cause a pro-inflammatory environment [26,27]. Burns et al. [8], report higher PSA levels in older patients with IBD than their age-matched controls, which may reflect underlying prostatic inflammation and/or injury. Moreover, the inflammatory state of IBD is mediated through pro-inflammatory cytokines, including interleukin 6 (IL-6). In fact, IL-6 receptors are upregulated in PCa cells and IL-6 plays a trophic role in cancer growth [28,29]. Indeed, we and others have shown that blood levels of IL-6 and its receptor are elevated in patients with metastatic PCa [30]. Moreover, they improved the predictive accuracy of standard predictive tools in patients treated with radical prostatectomy for non-metastatic PCa [31]. C- reactive protein (CRP), an acute phase reactant that is elevated in IBD, was also associated with higher PSA levels [32–34]. In fact, higher CRP levels were found to correlate with PCa resistance to treatment and poorer survival [34,35].

### Immunosuppressive medications

Furthermore, the use of immunosuppressive medications that control IBD may increase the risk of extra-intestinal cancers such as haematological malignancies (non-Hodgkin lymphoma) and skin cancers [36–39]. IBD immunosuppressive medications may be directly related to carcinogenesis by causing direct DNA damage, diminishing the immune surveillance over cancerous cells, or promoting chronic infections within tissues, which may induce aberrant cellular growths owing to chronic inflammatory status with associated oxidative stress [18,36,38].

### Exposure to medical personnel

An additional possible explanation for the higher PCa detection rates in patients with IBD is their frequent exposure to medical personnel, including more clinical examinations, such as DRE before colonoscopy procedures, and the periodic investigations requested for patients with IBD [17]. Whether this is merely an over diagnosis or results in detecting clinically significant tumours needs further evaluation [40,41].

### Prostate cancer considerations

While all included studies investigated the risk of developing PCa in the setting of IBD, none have examined the risk of high-risk PCa. Burns et al. [8] have concluded that IBD may be associated with an increased risk of clinically significant PCa (Grade Group ≥2). Alternatively, another report showed that patients with IBD were likely to have lower-risk PCa, but this study did not evaluate the risk of developing PCa among patients with IBD and, accordingly, was not included in the present review [42].

No clear association has been concluded about the survival outcomes in patients with PCa with IBD. Multiple studies reported serious bowel toxicities of pelvic radiation in patients with IBD [42,43]. Therefore, the restricted use of certain anti-neoplastic medications or radiotherapy may affect the overall survival of patients with PCa with IBD.

### Limitations and strengths

The present review is limited by the small number and observational nature of the included studies. The heterogeneity of the currently available data precluded a formal meta-analysis to estimate the effect of IBD on the risk of PCa. Nevertheless, we shed some light into an under-studied field in the literature. Understanding the association between IBD and PCa is crucial to identify patients at higher risk of PCa. It also aids in defining new screening thresholds for patients with IBD.

## Conclusion

Patients with IBD appear to have a higher risk of developing PCa, especially in patients with UC. These data support early PCa screening/early detection strategies in patients with UC. Data on the risk of PCa in patients with CD are inconclusive. Further studies are needed to elaborate on the association between IBD and PCa risk to help in patient counselling, treatment planning, and follow-up scheduling. The future holds promise to recognise a potential common molecular pathway between IBD and PCa, which may help identifying a targetable mechanism changing the treatment paradigm of patients with IBD and helping patients affected by both.
